# PeerPub: A Device for Concurrent Operant Oral Self-Administration by Multiple Rats

**DOI:** 10.1523/ENEURO.0241-24.2024

**Published:** 2025-01-08

**Authors:** Paige M. Lemen, Jie Ni, Jun Huang, Hao Chen

**Affiliations:** Department of Pharmacology, Addiction Science and Toxicology, University of Tennessee Health Science Center, Memphis, Tennessee 38163

**Keywords:** addiction, behavior, operant conditioning, drug, rat, self-administration

## Abstract

The social environment has long been recognized to play an important role in substance use, which is often modeled in rodents using operant conditioning. However, most operant chambers only accommodate one rodent at a time. We present PeerPub—a unique social operant chamber. PeerPub employs touch sensors to track the licking behavior on drinking spouts. When the number of licks meets a set reinforcement schedule, it dispenses a drop of solution with a fixed volume as a reward at the tip of the spout. A radio frequency identification (RFID) chip implanted in each rat’s skull identifies it throughout the experiment. The system is managed by a Raspberry Pi computer. We evaluated PeerPub using Sprague Dawley rats in daily 1 h sessions, where supersac (a glucose and saccharin solution) was provided under a fixed-ratio five schedule. We discovered that male rats consumed more supersac in dual rat conditions compared with single rat conditions. These findings illustrate PeerPub’s effectiveness in modeling the interaction between motivated behavior and social context. We expect devices like PeerPub will help highlight the role of social environments in substance use disorder phenotypes. All computer code, 3D design, and build instructions for PeerPub can be found at http://github.com/nijie321/PeerPub.

## Significance Statement

Social environments significantly influence food and drug consumption, but traditional operant chambers limit the study of these effects by accommodating only single animals. PeerPub, a novel social operant chamber, addresses this gap by enabling simultaneous oral operant drug self-administration in multiple rats. Using radio frequency identification and touch sensors, PeerPub tracks individual licking behaviors and delivers appropriate rewards for each individual. Testing with Sprague Dawley rats revealed that social interactions influence consumption behaviors, with male rats consuming more in social settings. PeerPub offers a powerful tool for studying the interplay between social context and motivated behavior, providing valuable insights into substance use disorder phenotypes and aiding in the development of effective prevention and treatment strategies.

## Introduction

The social environment is an important factor for human and rodent behavior. Social interaction plays a role in the perceived palatability of food or drink in humans (Herman et al., 2003; Robinson and Higgs, 2012; Leng et al., 2017; Suwalska and Bogdański, 2021). For example, social pressure can either increase (Pliner and Mann, 2004) or decrease (Paxton et al., 1999) the consumption of food. Additionally, there is strong evidence that social factors play multifaceted roles in substance use disorder (SUD). Individuals may be more likely to use drugs in the presence of other drug users (Gardner and Engel, 1971; Valente et al., 2007; Strickland and Smith, 2014). Alternatively, social relationships can promote recovery from SUD by functioning as a strong support network (Dobkin et al., 2002; Terrion, 2013; Pettersen et al., 2019).

Social environment is critical, but often neglected, when translating findings from basic studies to the prevention and treatment of SUD (Heilig et al., 2016; Venniro et al., 2020). Operant drug self-administration allows an animal to learn the association between an action (e.g., lever pressing, nose poking, or spout licking) with the delivery of a reward. While most operant studies were conducted using isolated animals, some have investigated the role of the social environment. For example, rats tested with a drug-experienced partner were faster to initiate cocaine self-administration (Gipson et al., 2011; Smith et al., 2014) and social learning-facilitated nicotine self-administration (Chen et al., 2011; Wang et al., 2016; Han et al., 2017). In contrast, rats tested with a drug-naive partner decreased cocaine SA (Smith, 2012; Strickland and Smith, 2014). Social environments have also been shown to have a protective effect on the risk of developing SUD-like behavior (Venniro et al., 2018).

Here, PeerPub is described—a device that allows multiple rats to learn oral operant drug self-administration simultaneously in the same chamber. PeerPub uses touch sensors to record licking events. These events are assigned to individual rats, based on the unique radio frequency identification (RFID) chip embedded under the skin on top of each rat’s head. A drop of solution is delivered to the tip of the spout when the number of licks meets the criteria of a reinforcement schedule. Additionally, data collected from PeerPub allow the analysis of lick microstructure, which provides insights into the subjective experience of the rodents consuming the drugs (Lin et al., 2013). We also designed an optional camera module. The image or video data provide insights into the pattern of social interactions during operant conditioning. PeerPub was tested using Sprague Dawley rats by allowing them access to Supersac (a solution containing glucose and saccharin). We found a significant interaction between social environment and sex on supersac oral self-administration.

## Materials and Methods

### Overview of the system

**Figure 1.**
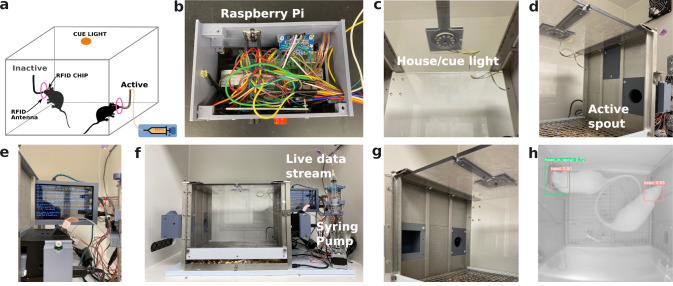
PeerPub design setup. ***a***, Drug self-administration chamber setup for recording drug intake and licking microstructure of two rats simultaneously. ***b***, The Raspberry Pi computer that collects data in real time, placed on the side of the operant chamber. ***c***, The cue light that flashes each time a reward is given by the pump. ***d***, The “active” spout and holder that is attached to a syringe and pump containing a substance for oral self-administration. ***e***, The screen of a Raspberry Pi computer attached to the operant chamber. This shows data being collected in real time. ***f***, Full setup of all parts of the oral drug self-administration chamber and attached parts. ***g***, The “inactive” spout is the control spout used to compare licks between spouts with and without drug access. The water spout is an optional addition for sessions that run for a long time and animals need access to water. The spout holder is removable and can be replaced with more chamber walls. ***h***, View of the operant chamber from PeerPub’s camera. PeerPub has the option to place a camera above the chamber to record both the spout and social behavior between rats during self-administration. Images are analyzed using convolutional neural networks to identify the head, nose, tail, tail base, and entire body. Locations of the bounding boxes, recorded as *x* and *y* coordinates, are collected in a CSV file.

PeerPub is an operant chamber that records the licking behavior of multiple rats simultaneously (Fig. 1). The overall design has similarities to our previous device (Longley et al., 2017) but accommodates multiple rats. Each PeerPub device has two stainless steel rodent lickets (with a stainless steel ball at the end). One of the spouts is designated to be active and the other one is inactive. These spouts are installed in two identical 3D-printed spout holders. Each holder connects the spout to a unique channel on a capacitive touch sensor, which records the timing of each lick with millisecond time resolution. The faceplate of the spout holder contains an antenna for the RFID system. Each rat has an RFID chip embedded on top of its head. The physical design of the spout holder forces the rat to pass the RFID antenna before its tongue can reach the spout. Each lick is assigned to the RFID detected immediately prior to the lick (with a maximal delay of 3 s). Upon the completion of a reinforcement schedule on the active spout, a drop of solution (i.e., the reward) is pushed to the tip of the spout via a syringe driven by a step motor (i.e., a pump). Licking on the inactive spout has no consequences, but all the licks are recorded. The system is controlled by a Raspberry Pi (RPi) computer. A set of RFID fobs with their IDs stored in the software is used to set the session length and reinforcement schedule (e.g., fixed or variable ratios) and start each test session. Data are saved in the microSD card of the RPi and can be transferred wirelessly to a remote server upon the completion of each session. Both the spout holders and the case of the RPi are 77 mm in width. They can be installed in standard commercial rat operant chambers. PeerPub allows for the addition of a water bottle spout (Fig. 1*g*) to provide supplementary water. However, the experimental design for the current study did not use this option because sessions were only 1 h in length.

### Hardware

The RPi is a credit card–sized single-board computer. The RPi 3 (Model B) was used in this project. Each RPi has 40 general-purpose input/output (GPIO) pins. These pins are the interface between the RPi and other electronic modules, such as touch sensors (input), LED lights (output), and step motors (output).

The EM4100 RFID system was used because of the availability of inexpensive readers with sensitivity sufficient for this experiment’s design. The injectable RFID chip was 2 × 8 mm, each emitting a unique hexadecimal code when placed in the vicinity of the antenna. The antenna guarding the active spout was configured to output the encoded unique ID as an 8-digit hexadecimal code, while the inactive antenna produced a 10-digit hexadecimal code. This configuration was accomplished using Windows software provided by the manufacturer. This setup allowed the program to distinguish the licking behavior emitted from the spouts. A circular antenna with a diameter of 45 mm was used. The antenna is placed vertically behind the spout holder’s faceplate, forcing the rat to place its head inside the antenna loop when licking the spout. The opening of the faceplate was further adjusted to ensure that the rat’s skull was almost touching the upper edge of the opening, where the RFID on the skull would generate the strongest signal. Given that the opening is sufficiently narrow to accommodate only one rat’s head at a time, the likelihood of simultaneous detection of multiple RFIDs is effectively eliminated, preventing any potential signal collision.

We further conducted a thorough evaluation of potential RFID signal collisions in both spatial and temporal dimensions. Spatially, our findings indicate that the RFID tags must overlap by >4 mm to cause the system to fail in detecting the unique ID of each chip. This overlap distance is impossible to achieve when the RFIDs are embedded in the rats’ heads. In terms of temporal resolution, we assessed the system’s performance by sequentially dropping two RFID tags through the antenna loop. We found that the system reliably detects the second RFID tag if it passes through the loop >0.25 s after the first tag. This time interval is sufficiently large to prevent RFID collision due to the rapid movement of the rats, ensuring that each rat is readily detected without interference. In addition, a set of RFID chips embedded in plastic fobs was used to start each session with specific configurations. These fobs allowed lab personnel to start the session without using a keyboard.

A NeoPixel RGB LED ring is a small electronic component that consists of programmable color LEDs arranged in a closely spaced circle. Each LED can be controlled by outputting a tuple of three eighth-bit values ranging from 0 to 255 to control color and brightness. A NeoPixel ring with 12 LEDs was used as house and cue lights. The house light is turned on for a time period between 9:00 P.M. and 9:00 A.M. and is turned off the rest of the time (rats were housed in reverse light-cycled rooms). The LED ring was programmed to flash for 0.5 s when a reward was delivered. This acted as a cue for drug reward.

A NEMA 17-size hybrid bipolar stepper motor was attached to a rectangular metal channel, along with several 3D-printed mount pieces for the syringe tube, for positioning purposes. A DRV8834 low-voltage stepper motor driver connects the RPi and the stepper motor to allow the configuration of precise steps and rotation. The motor has a 1.8° step angle, which allows for precise control with 200 steps per revolution. This is complemented by the driver’s capacity to handle up to 2 A output current per coil, which ensures that the motor can apply 3.7 kg of force at a distance of 1 cm from the pivot point, translating to strong and accurate positioning capabilities. To make sure the stepper motor’s power is enough to drive the syringe precisely, the distance between the syringe and the pump can be adjusted, which can modify the torque accordingly to rotate the pump rod promptly and accurately. The less distance the more torque. The distance between the center of the syringe and pump rod is 27.7 mm in the current experimental setting. The pump design was based on an open-source design (Varnon, 2016). Two push buttons (forward and backward) were connected to the RPi via GPIO to allow manual syringe pump adjustment. After each session, the plunger will be at a higher position; it is necessary to reposition it so the syringe can be reloaded before a new session. In addition, two limit buttons were mounted to the side of the channel for the purpose of repositioning the plunger when the syringe reached the end position. The software was programmed to send an alert message via the wireless network to a communication application (slack) when this happened, to alert the technician that the syringe needed to be refilled. The forward button will advance the syringe while it is being held in place. In contrast, the back button will automatically lower the plunger until the edge of the plunger hits the limit switch.

In order to ensure precise delivery of the designated reward volume (60 µl of supersac in this experimental context), a pump calibration process was integrated into the PeerPub system. Activation of this process is facilitated through an additional RFID chip embedded within a plastic fob. This procedure involves collecting five deliveries of the designed volume, totaling 300 µl (60 µl × 5 = 300 µl). This procedure is iterated three times, resulting in a cumulative reward volume of 900 µl. PeerPub prompts the operator to input the actual amount dispensed during each iteration and subsequently adjusts the pump settings to achieve the intended volume accurately. Once a pump is calibrated, it is stable for many months. It is important to execute the calibration process regularly to ensure the precision of the reward given to the rats.

The Adafruit Capacitive Touch Sensor Breakout (MPR121) was used to record licking activities. The default electrode sample interval is 16 ms, and it is programmable to a maximum interval of 128 ms. The default 16 ms interval is used in PeerPub, to ensure maximal accuracy in timing the licks. The active and inactive spouts were connected via separate electric wires to the sensor. Only 2 of the 12 available channels were used, one for each licking spout.

A 5 inch display was connected to the RPi for display real-time data and debugging. To fit these sensors on the side of the chamber, a 3D-printed case was designed to fit the RPi and other electronic components connected to the GPIO pins. The case was designed to fit in the slots of standard commercial rat operant chambers, but the design can be easily adjusted. The RFID readers were connected to a powered external USB hub to ensure sufficient sensitivity. A 3 A power adapter was used to power the RPi. The touch sensor that records licking activities also allows researchers to record and analyze the lick microstructure for each rat.

### Software and code availability

Python 3 was used to program the operant sessions via third-party libraries for the GPIOs. The gpiozero and RPi.GPIO libraries were used to control external input/output modules. The main program asks the user to enter a specific type of session, which can be provided by scanning the specific RFID fobs without using a keyboard. The RFIDs implanted in the rats are then scanned. The main program then spawns a separate thread to handle licking behavior logics while the main thread is kept alive to record rats’ RFIDs when they poke their head into the spout holder. The second thread counts the number of licks on the active and inactive spouts associated with each and delivers rewards according to the reinforcement schedule. The program was written in an object-oriented fashion. Software to run PeerPub and for 3D printing of parts are available at http://github.com/nijie321/PeerPub.

The Raspbian operating system was utilized without a graphical user interface to reduce computational overhead. All operations are done on the console, and colored prompt texts are used to provide behavior data in real time.

### Social interaction recording

An RPi camera was attached to the top of a rat operant chamber facing downward in order to record social interactions (Fig. 1*h*). We replaced the top of the operant chamber using a piece of clear plexiglass to allow for unobstructed viewing by the camera. Although the system is capable of recording videos, the camera was programmed to take one picture every 2 s to reduce CPU load.

### Validation of recorded licks

To validate the accuracy of PeerPub’s lick detection, we designed a spout holder with an open side wall to allow for video recording of the licking events. Using a cell phone camera capable of recording high-definition video at 240 frames per second, we recorded the licking behavior of two rats. The PeerPub LCD monitor, which displays each recorded licking event, was visible in the background of the videos. This setup enabled us to visually count the rat’s tongue contacts with the spout and compare them to the number of licks recorded by PeerPub.

### Validation of reward delivery

To validate the amount of liquid dispensed per reward, 10 empty microcentrifuge tubes were weighed, and then each was positioned beneath the active spout to collect the amount of liquid received for one reward triggered manually (water was used as the reward). These tubes were then weighed again. The difference in weights is then converted to the volume of reward (60 µl of water = 0.06 g).

### Lick microstructure analysis

In rodent studies, a “lick cluster” refers to a series of licks that occur in rapid succession, often during the consumption of a liquid reward. We define a cluster as sustaining licks until a pause of 1 s. In addition to the number of clusters, which often is correlated with the volume of consumption, we also analyze interlick interval (ILI, i.e., the average time lapse between individual licks) and cluster size (i.e., the average number of licks within lick clusters). These measurements help to characterize patterns of consumption and offer insights into the subjective value of the reward (Vajnerová et al., 2003; Weiss and Di Lorenzo, 2012; Lin et al., 2013).

### Statistical data analysis

Data were analyzed using the R programming language. Mixed factorial ANOVA was used to examine the effects of the treatment group (single vs duo in the operant chamber), session, and sex, on operant behaviors, including intake, number of rewards, and licks on the spouts. Session was a within-subject variable, and other factors were between-subject variables. Before ANOVA analysis, the number of licks was converted to a log scale. Data were presented as mean ± standard error. Lick microstructure data were analyzed similarly using mixed factorial ANOVA.

Images of rats were analyzed using YOLOv5, a convolutional neural network with state-of-the-art object detection performance (Ultralytics, 2023). To use YOLO to extract the locations of different body parts of the rat from the images, we first annotated different objects, including the rat, head, nose, tail base, tail, active spout, inactive spout, and head in spout with bounding boxes. These annotations are considered the “ground truth” against which predictions are compared. We fine-tuned the network weight of default YOLOv5 for 318 epochs on a high-performance computing cluster using 172 labeled images and then tested against a new set of 41 labeled images that were not used in training. We assess the accuracy of bounding box prediction with the intersection over union (IoU) metric, which is a measure of overlap between the predicted bounding box and the ground truth. A higher IoU means a better prediction, and 50% is set to determine whether a detection is considered correct. The average classification accuracy, precision, and recall are 88, 94, and 87%, respectively. We then used this network to detect objects in all images captured during self-administration sessions.

R programming language was used to analyze the location of objects from captured images to infer social interaction between rats. We are particularly interested in the distance between the heads of the two rats and their relative distance with the two drinking spouts during the self-administration sessions. We normalized the location of the active spout across all images to their average position to enable comparison across experimental chambers and sessions to compensate for different relative camera placement. This step also adjusted the location of other objects present in the images (e.g., heads). Pearson’s correlation was used to examine the relationship between head-to-head distance and the average head-to-spout distance of both rats.

### Experimental design

The effect of social isolation and supersac self-administration were examined using young adult Sprague Dawley rats (postnatal day approximately 55 at the onset of the experiment). Rats were placed in either a cage alone (single) or paired with one other cagemate (duo), with males = 12–16/group and females = 10–14/group. All rats went through a 10 d habituation phase to make sure they were well acquainted with their cagemate or isolation (depending on their experimental group) before beginning the self-administration protocol. Both housing and chamber environment (paired or isolated) remained matched throughout the study. The protocol consisted of a brief 4 h water deprivation before the onset of operant training and then 1 h oral supersac self-administration in operant chambers equipped with PeerPub for eight sessions (one water deprivation/session/day).

Supersac (3% glucose plus 0.0125% saccharin in water) is a highly appetitive solution and elicits strong oral consumption in rats (June and Gilpin, 2010; Chen et al., 2011). A fixed-ratio five (FR5) reinforcement schedule was used for supersac reward (60 µl each). The total number and timing of rewards and the licks on the active and inactive spouts were recorded for each rat. Body weights were recorded before each session. Licking on the inactive spout resulted in no reward or punishment and was used as a control. Presenting both an active and inactive option in chambers allows for the validation of operant conditioning and the preference of the chosen substance as a reward. It also allows the testing of whether a substance will maintain the self-administration protocol by the significant difference between rats responding to the substance versus responding to the negative control (O’Connor et al., 2011).

## Results

We evaluated the accuracy and reliability of PeerPub. We found RFID tags were detected exclusively when they traverse the entrance to the spouts, specifically along the upper edge of the entrance (Fig. 1), which occurs when rats insert their heads to access the spout. RFID tags do not interfere with each other unless they physically touch or pass through the entrance within 0.25 s of each other, which does not occur when the tags are implanted in rats. Using videos recorded at 240 FPS, we analyzed 10 licking bouts where the movement of the tongue was not obstructed from the view and compared those recorded by PeerPub. We found the mean accuracy of licks recorded by the touch sensor was 92.4 ± 5.7%.

**Figure 2.**
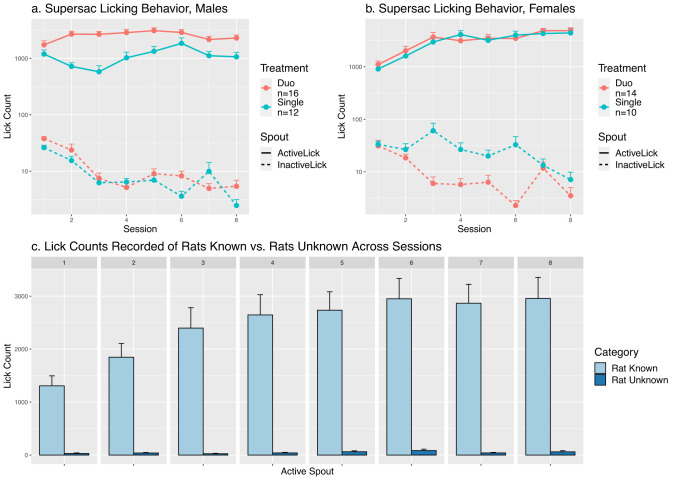
Licking behavior of rats for the active and inactive spouts in log scale. The line type indicates the spout at which the licking occurred (solid lines are licks on the spout that dispensed supersac—the “ActiveLick” spout. Dashed lines are licks on the control spout with no supersac attached—the “InactiveLick” spout). Rats consistently licked the active spout more than the inactive spout (*F*_1,1209_ = 448.6, *p* = 2 × 10^−16^) indicating a preference for self-administering supersac. ***a***, Number of licks per session in males, single versus duo and active spout licks versus inactive spout licks. Duo male rats (self-administration with cagemate) licked the active spout significantly more than the single group (*F*_1,23_ = 6.5, *p* = 0.018). ***b***, Number of licks per session in females, single versus duo and active spout licks versus inactive spout licks. No significant differences between treatment groups in licking behavior were observed for female rats (*F*_1,22_ = 0.019, *p* = 0.89). ***c***, Lick counts recorded of rats with RFID versus RatUnknowns across sessions. PeerPub assigns each lick to a unique RFID. On rare occasions, the antenna did not detect the RFID, resulting in licks being recorded as “RatUnknown.” These unassigned licks comprised only 1.9% of the total licks across all sessions.

PeerPub assigns licking events where no RFID tag was detected to “RatUnknown.” These unassigned licks constituted 1.9% of the total licks (Fig. 2*c*). We measured the volume of 10 consecutive rewards, yielding a mean of 59.8 ± 4.9 µl, almost identical to the expected 60 µl. Together, these data indicate that PeerPub is highly accurate in detecting RFIDs, recording licking events, assigning licks to the appropriate rats, and delivering rewards.

We used PeerPub to evaluate the effect of social interaction on the consumption of supersac. For the number of licks (Fig. 2), we found all rats, regardless of sex or treatment, licked the active spout significantly more than the inactive spout (*F*_1,1209_ = 443.1, *p* = 2 × 10^−16^). For example, mean lick counts were 1,785.9 ± 277.7 on the active spout and 2.8 ± 0.72 on the inactive spout for Session 8. These data indicate licking is motivated by supersac. We found a significant sex difference in the number of active licks (*F*_1,45_ = 5.8, *p* = 0.20). Across all sessions, social interaction increased the number of licks on the active spout in males (*F*_1,23_ = 6.56, *p* = 0.017) but not in females (*F*_1,22_ = 0.015, *p* = 0.91). Furthermore, the active lick count in single females was statistically similar to that in duo males (*p* = 0.55).

**Figure 3.**
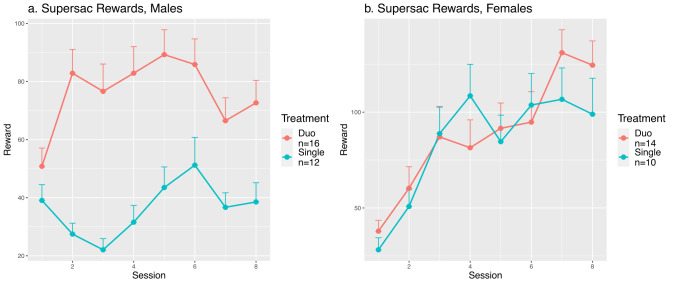
Impact of chamber social conditions on supersac intake in Sprague Dawley rats. ***a***, Number of rewards given per session in males; single versus duo. Male rats that underwent self-administration with their cagemate (duo) took more supersac than rats alone during self-administration (single) during these eight sessions (*F*_1,23_ = 7.35, *p* = 0.012). ***b***, Number of rewards given per session in females; single versus duo. No significant differences in intake were observed between treatment groups for female rats (*F*_1,22_ = 0.14, *p* = 0.72).

We found a significant sex difference in supersac consumption (*F*_1,46_ = 22.3, *p* < 2.2 × 10^−5^). The social environment significantly impacted supersac consumption in male rats (*F*_1,23_ = 7.35, *p* = 0.012), with single and duo rats consuming 0.72 ± 0.06 and 1.45 ± 0.08 mg/kg per session, respectively. However, social environments did not affect supersac intake in females (*F*_1,22_ = 0.14, *p* = 0.72, 2.54 ± 0.23 for single and 2.78 ± 0.22 mg/kg for duo). These data revealed a notable difference in the effect of social environment between sexes (Fig. 3).

**Figure 4.**
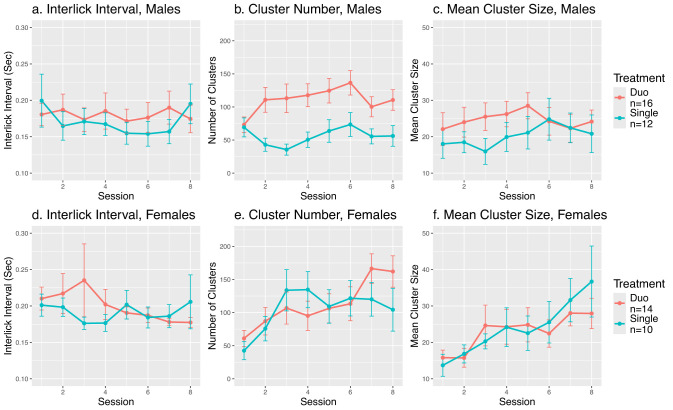
Lick microstructure of males’ and females’ analyses between treatment groups. ***a***, Interlick interval in males. Treatment group difference was not significant (*F*_1,24_ = 0.12, *p* = 0.73). ***b***, Number of lick clusters in males. Lick cluster number was significantly higher for duo rats (*F*_1,24_ = 11.44, *p* = 0.0025). ***c***, Mean cluster size in males. There were no significant differences between groups (*F*_1,24_ = 0.99, *p* = 0.33). ***d***, Interlick interval in females. Treatment group difference was not significant (*F*_1,21_ = 0.19, *p* = 0.67). ***e***, Number of lick clusters in females. There were no significant differences between groups (*F*_1,22_ = 0.09, *p* = 0.77). ***f***, Mean cluster size in females. There were no significant differences between groups (*F*_1,21_ = 0.02, *p* = 0.89).

For lick microstructure, social context did not significantly change ILI in males (*F*_1,24_ = 0.12, *p* = 0.73) or females (*F*_1,21_ = 0.19, *p* = 0.67, Fig. 4*a,d*). Duo males had significantly more lick clusters than single males (*F*_1,24_ = 11.44, *p* = 0.0025, Fig. 4*b*, 55.81 ± 4.80 for single and 110.80 ± 6.17 for duo groups). However, the number of lick clusters was similar between single (105.29 ± 9.30) and duo (112.32 ± 8.17) females (*F*_1,22_ = 0.09, *p* = 0.77, Fig. 4*e*). The social environment did not affect the mean cluster size in either sex (males *F*_1,24_ = 0.99, *p* = 0.33; females *F*_1,21_ = 0.02, *p* = 0.89; Fig. 4*c,f*). In addition, across the sessions, there was a progressive reduction in the latency to lick the active spout (*F*_7,355_ = 17.54, *p* < 2 × 10^−16^). The increased number of clusters in duo males compared with single males is in agreement with intake data. The lack of effect on ILI and cluster size indicates the subjective value of supersac was not affected by the social environment.

**Figure 5.**
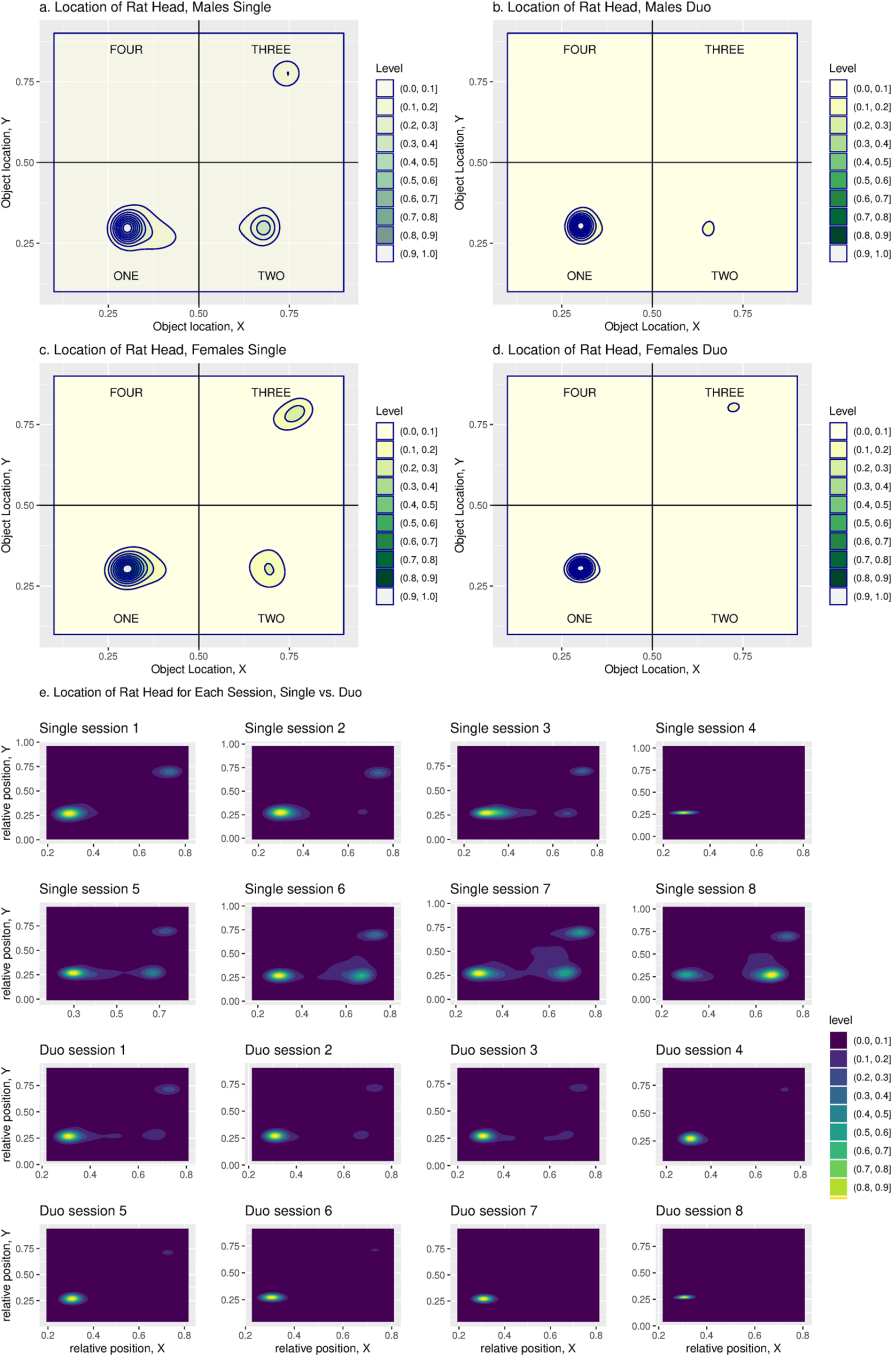
Density plots of rat head positions in the PeerPub operant chamber. The density plots illustrate the frequency distribution of the rats’ head positions within the operant chamber, as observed from a top-down perspective. For analytical purposes, the chamber is divided into four quadrants. The active spout is situated in the first quadrant, close to the origins of the *x*- and *y*-axes. Four experimental groups were evaluated, where (***a***) a single male rat, (***b***) two male rats, (***c***) a single female rat, and (***d***) two female rats were tested in eight daily sessions. The color gradient on the plots represents the density of head positions, with darker hues indicating regions where the rats’ heads were most frequently located. Contour lines demarcate the areas of equal density, thereby highlighting zones where the rats spent a significant amount of time across all eight sessions. (***e***) Density maps of rat head positions across individual sessions in the PeerPub operant chamber, single versus duo. The location of rats’ heads is displayed for each session across eight consecutive sessions in a bird’s eye view as a two-dimensional coordinate plane. The color levels represent the density of observations at each location within the operant chamber, with darker green to blue shades indicating areas where the rat’s head was more frequently located and lighter shades indicating less frequent locations. The *x* and *y*-axes represent relative positions within the chamber. The top row of plots shows the head positions for rats in single housing during each of the eight sessions. The bottom row shows the head positions of rats housed in pairs with their cage mates during each of the eight sessions.

Social interaction was analyzed using image data. Head positions were recorded as coordinates on an *x*–*y* plane in the operant chamber. Spatial distribution of head positions revealed distinct engagement patterns between single and duo groups, as evidenced by the density distributions (Fig. 5). In both sexes, density curves indicate a strong preference for the region containing the active spout (Quadrant 1). Single rats had a broader head location placement across quadrants, whereas duo rats stayed near the active spout (Fig. 5). Over successive sessions, a discernible trend emerged wherein duo group head locations consolidated, while single group locations dispersed more (Fig. 5*e*).

**Figure 6.**
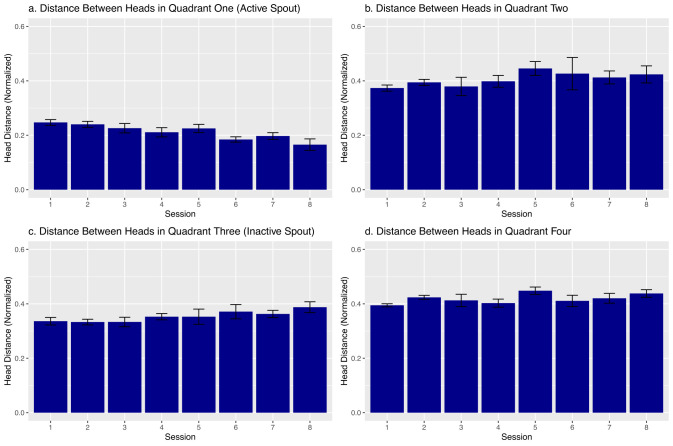
Distance between rats’ heads normalized across sessions 1 to 8. No significant sex differences were observed in head distance across quadrants or sessions, so data were combined for analysis. The mean normalized head distance was 0.21 ± 0.00099, 0.39 ± 0.0018, 0.34 ± 0.0030, and 0.42 ± 0.0031 units for Quadrants 1–4, respectively. A two-way mixed ANOVA revealed significant differences in head distance by quadrant (*F*_3,12_ = 46.23, *p* = 7.22 × 10^−7^) and a significant interaction between quadrant and session (*F*_21,99_ = 2.22, *p* = 0.0047). Subsequent analysis indicated that the effect of session on head distance was significant only in Quadrant 1 (*F*_7,33_ = 5.72, *p* = 0.0022). ***a***, Distance between rats’ heads shown in Quadrant 1. This quadrant is the bottom left quarter of the chamber as well as the location of the active spout. Distance between heads here gradually decreases over time. ***b***, Distance between rats’ heads shown in Quadrant 2. This quadrant is the bottom right quarter of the chamber and does not include any spouts. ***c***, Distance between rats’ heads shown in Quadrant 3. This quadrant is the top right quadrant and includes the location of the inactive spout. The distance here gradually increases over time. ***d***, Distance between rats’ heads shown in Quadrant 4. This quadrant is the top right quarter of the chamber and does not include any spouts.

Although these images cannot identify each rat, we calculated the distance between the heads of paired rats (Fig. 6). We found a progressive reduction of head distance in the quadrant containing the active spout across the sessions (Fig. 6*a*). No sex difference was seen in the head distance, and data were combined for subsequent analysis. The mean normalized head distance was 0.21 ± 0.00099, 0.39 ± 0.0018, 0.34 ± 0.0030, and 0.42 ± 0.0031 for Quadrants 1–4, respectively (*F*_3,12_ = 46.23, *p* = 7.22 × 10^−7^). There was also a significant interaction between the quadrant and session (*F*_21,99_ = 2.22, *p* = 0.0047). Further analysis found that the effect of the session on head distance was only significant in Quadrant 1 (*F*_7,33_ = 5.72, *p* = 0.0022).

**Figure 7.**
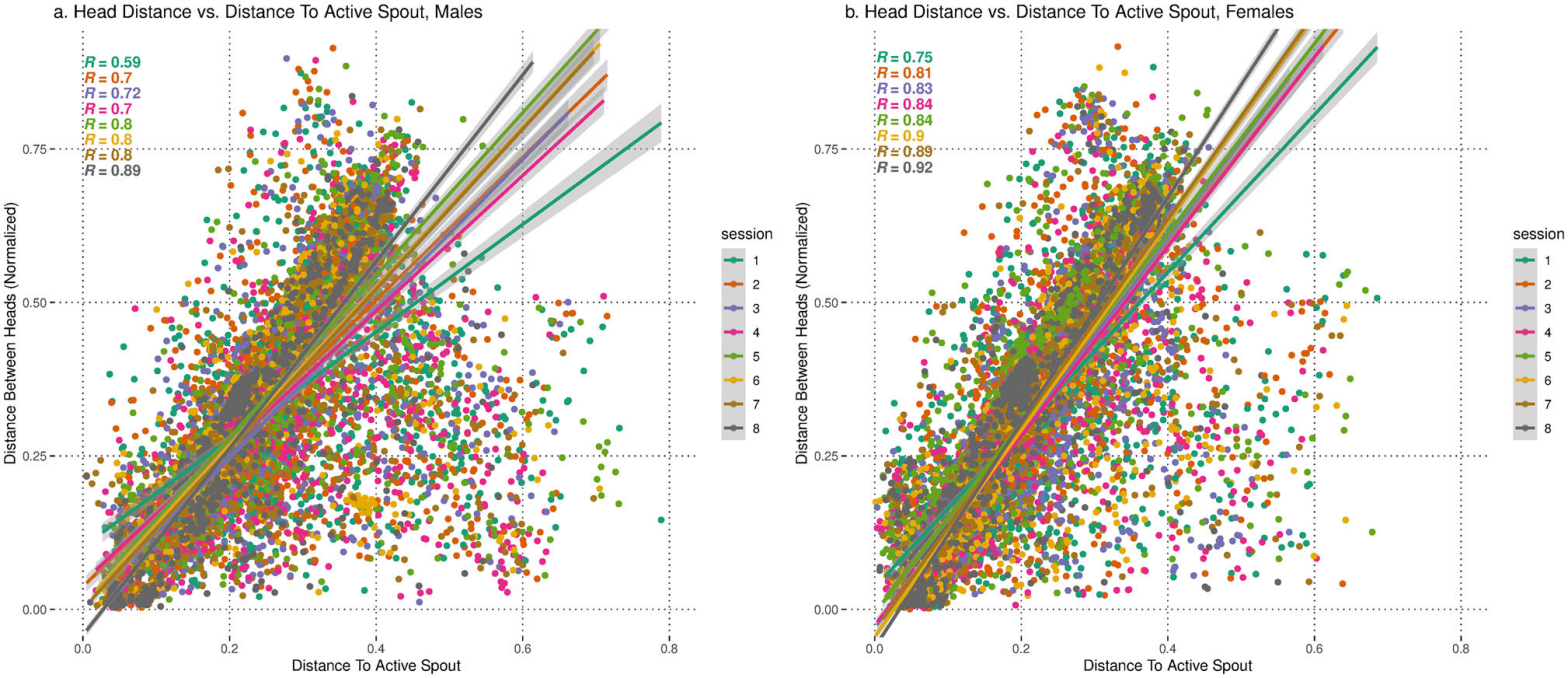
Correlation of normalized head distance and distance to active spout across sessions for males and females. Pearson's correlation coefficient assessing the linear relationship of normalized distance between rats’ heads versus distance to the active spout (ps < 2.2 × 10^−16^ for all) across sessions 1–8. ***a***, Pearson’s correlation for males. There was a positive correlation between the two variables, increasing across sessions, from *R* = 0.59 to *R* = 0.89. ***b***, Pearson’s correlation for females. There was a positive correlation between the two variables, increasing across sessions, from *R* = 0.75 to *R* = 0.92.

We found a strong positive correlation between head-to-head distance and the head-to-spout distance in both sexes (Fig. 7). This indicates that as one of the rats moves closer to the active spout, the other rat often follows. Furthermore, the correlation coefficient increases across sessions (*R* = 0.59 to 0.89 in males, *R* = 0.75 to 0.92 in females, ps < 2.2 × 10^−16^ for all correlations), suggesting stronger coordination between rats. The slope of the regression line increases across sessions (Fig. 7), indicating rats are getting closer to each other near the active spout over time.

## Discussion

PeerPub is a novel apparatus designed to enable the simultaneous oral operant substance self-administration of multiple rats. RFIDs embedded on top of each rat were used to track the identity of rats. We evaluated the performance of the detection of RFID, recording of licks, assignment of licks to rats, and reward delivery and confirmed their accuracy. We tested PeerPub by studying supersac oral self-administration. We found that males, but not females, showed higher supersac intake in the duo setup compared with the single rat condition, with lick microstructure analysis also showing a higher number of lick clusters. Using an optional camera module, we found, in both males and females, that rats in the duo group stayed almost exclusively in the vicinity of the active spout, while rats in the single group explored other parts of the chamber. These results collectively demonstrated PeerPub’s utility in examining oral self-administration in a social environment.

PeerPub is developed to model the misuse of substances by oral consumption (Sharp et al., 2021), where the social environment plays a critical role but is often ignored (Peitz et al., 2013; Heilig et al., 2016). Several approaches have been used to incorporate social environments into operant conditioning experiments. Because physical isolation is required to preserve the catheters used for intravenous drug delivery, operant chambers have been modified to isolate rats but permitting limited social contact via either plexiglass (Gipson et al., 2011), a mesh (Peartree et al., 2017), or several small windows (Han et al., 2017). However, voluntary oral consumption, often the predecessor of drug injection or snorting (Katz et al., 2011; Surratt et al., 2011), provides an opportunity to model drug use in more natural social settings. For example, the Rat Park experiments found that rats living in a social environment were less likely to consume morphine orally, as opposed to rats living in an isolated environment (Alexander et al., 1978). These pioneering experiments, however, did not use operant conditioning procedures. Our design provides a novel paradigm, where the operant conditioning of two rats can be carried out simultaneously in the same chamber, allowing rats with unrestricted social contact.

We found that male rats tested with a peer drank significantly larger amounts of supersac than those tested alone. These findings support prior literature that suggests rats can learn behaviors through peer influence (Zentall and Levine, 1972; Wang and Chen, 2014). In addition, other studies also suggested that social behavior itself can be a reward (Venniro and Shaham, 2020; Navarrete et al., 2024). Similar to drug use, the eating habits of humans are shaped by social contexts (Higgs and Thomas, 2016). Social influences, such as family, peers, and religion, play a role in the likelihood of initial and continued use of substances, as well as the risk of developing an SUD (Dew et al., 2007).

Different from males, we found females in the single and duo groups consumed a similar amount of supersac. This absence of difference is likely due to the lick in single females reaching the maximum amount, thus masking the potential effect of the social environment. In supporting this idea, we found the number of active licks in single females was statistically similar to those by males in the duo group (Fig. 2). Therefore, future experiments studying the social effect on reward should avoid providing a reward with very high appetitive value.

PeerPub records each lick with a precise timestamp, enabling analysis of licking microstructures. The number of clusters is often directly associated with consumption, as found in our results (Fig. 4*b,e*). The size of the lick cluster and ILI offer an opportunity to measure the subjective value of the reward (Dwyer, 2012), neither was affected by the social environment (Fig. 4*c,f*). This could be due to the very high palatability of supersac (Valenstein et al., 1967) to rats masking the enhancement by social environment.

The optional camera module of PeerPub allows for a detailed examination of social dynamics. The density of head location revealed that duo rats spent increasingly more time near the active spout compared with the single group, aligning with their higher intake. Although social context did not significantly impact intake in females, it did affect their position similarly in males, further supporting a ceiling effect on intake. Together, these data illustrate the nuanced nature of the social environment on consumption, beyond resource limitations or stress (Brown and Grunberg, 1996).

PeerPub facilitates the quantification and analysis of subtle social behaviors, revealing patterns and trends that are difficult to detect manually, thereby enhancing our understanding of social interactions. However, the device requires programming and engineering skills for assembly, and the image analysis does not fully utilize the latest tools using artificial intelligence. Additionally, PeerPub is limited to recording oral self-administration. Future improvements could address these limitations.

In summary, we demonstrated that PeerPub is a reliable device for duo rat oral operant self-administration. The data from supersac study revealed that the effect of the social environment can be different between males and females and may also be influenced by the palatability of the drug. The complex interaction between social environment and reward revealed by PeerPub supports its use in studies of substance abuse research.

## Synthesis

Reviewing Editor: Christie Fowler, University of California Irvine Decisions are customarily a result of the Reviewing Editor and the peer reviewers coming together and discussing their recommendations until a consensus is reached. When revisions are invited, a fact-based synthesis statement explaining their decision and outlining what is needed to prepare a revision will be listed below. The following reviewer(s) agreed to reveal their identity: NONE.

The revised manuscript successfully addresses previous concerns regarding statistical analysis, technical validation, and methodological clarity. The authors have strengthened the work by adding rigorous validation of the RFID and lick detection systems, implementing appropriate statistical approaches, and expanding the discussion of translational implications.
